# Whole-Genome Sequencing and Pathogenic Characterization of a *Pasteurella multocida* Serotype A Isolate from a Case of Respiratory Disease in Tan Sheep

**DOI:** 10.3390/microorganisms14010154

**Published:** 2026-01-09

**Authors:** Yuxi Zhao, Pan Wang, Yuqiu Yang, Yarong Xu, Jiandong Wang

**Affiliations:** 1 Institute of Animal Science, Ningxia Academy of Agricultural and Forestry Sciences, Yinchuan 750002, China; zyxwpc18214@gmail.com; 2 School of Animal Science and Technology, Ningxia University, Yinchuan 750021, China; wp_1112@163.com (P.W.); yangyuqiu0729@163.com (Y.Y.); xyr_0305@163.com (Y.X.)

**Keywords:** tan sheep, *Pasteurella multocida*, strain P6, respiratory disease, pathogenicity, whole-genome sequencing, comparative genomics

## Abstract

Tan sheep are a characteristic and economically important local breed in the Ningxia Hui Autonomous Region of China, where respiratory diseases continue to pose challenges to animal health and production. In this study, a *Pasteurella multocida* strain (P6) was isolated from the lung tissue of a single Tan sheep presenting with severe and fatal respiratory disease, and subjected to case-based genomic and pathogenic characterization. The isolate was identified as capsular serotype A based on biochemical profiling, 16S rRNA gene sequencing, kmt-1 PCR, and capsular typing. To provide supportive evidence of virulence potential, a murine infection model was employed, in which P6 induced acute clinical signs and severe pulmonary lesions, including congestion, edema, hemorrhage, and fibrinous inflammatory exudation. Whole-genome sequencing revealed that strain P6 possesses a 2,289,251 bp genome with a GC content of 40.2%, encoding 2155 predicted genes and multiple mobile genetic elements, including genomic islands, prophages, transposons, and a CRISPR locus. Phylogenetic analysis based on seven housekeeping genes placed P6 in close relationship with strains 166CV and 103220, distinct from several rodent- and avian-derived isolates. Functional genomic analyses identified numerous genes associated with carbohydrate metabolism, secondary metabolite biosynthesis, host–pathogen interaction, virulence-related functions, and antimicrobial resistance. Comparative genomic analysis with the reference strain PM70 indicated a largely conserved functional framework, accompanied by a significant enrichment of mobilome-associated genes, suggesting enhanced genomic plasticity. Overall, this study provides a descriptive genomic overview of a *P. multocida* isolate associated with respiratory disease in Tan sheep and highlights its genetic features and potential adaptive capacity, while acknowledging the limitations inherent to a single-case investigation.

## 1. Introduction

*Pasteurella multocida* (*P. multocida*) is a Gram-negative, facultatively anaerobic coccobacillus that serves as both a commensal organism of the upper respiratory tract and an opportunistic pathogen capable of causing severe diseases in a wide range of domestic and wild animals, as well as in humans [1,2]. This versatile bacterium has been recognized for over 135 years since its first isolation by Louis Pasteur in the 1880s as the causative agent of fowl cholera [3]. Recent reports from 2024 underscore its continued significant impact across species, including outbreaks of acute pneumonic pasteurellosis in camels [4]. In livestock, *P. multocida* is responsible for economically devastating diseases, including hemorrhagic septicemia in ruminants [5], fowl cholera in avian species [6], progressive atrophic rhinitis in swine, and pneumonic pasteurellosis (associated with markers of apoptosis and necroptosis in cattle) across multiple host species [2,7,8].

In sheep, *P. multocida* is frequently associated with respiratory diseases, particularly pneumonic pasteurellosis, which can occur as a primary infection or secondary to viral respiratory insults and environmental stressors [1,9,10]. The pathogen is commonly classified into five capsular serogroups (A, B, D, E, and F) and sixteen lipopolysaccharide (LPS) serovars [11], with serotype A being predominantly associated with respiratory diseases in sheep, goats, and cattle [12]. Field studies have demonstrated that *P. multocida* capsular type A isolates from sheep with respiratory diseases are among the most prevalent pathogens, with LPS genotypes L3 and L6 being particularly common in ovine infections [13,14].

The Tan sheep (*Ovis aries*), indigenous to the Ningxia Hui Autonomous Region of China, represents one of the most economically important local sheep breeds in the country, renowned for its tender meat, unique flavor, and high-quality lamb wool [15]. However, the intensive development of Tan sheep farming in Ningxia and adjacent regions has been challenged by various health issues, including respiratory diseases that result in significant mortality and economic losses. The traditional closed breeding systems prevalent in many Ningxia sheep farms, characterized by simple housing structures and limited environmental control, create conditions conducive to respiratory pathogen transmission, particularly during winter months when ventilation is reduced.

The advent of whole-genome sequencing (WGS) and comparative genomics has revolutionized our understanding of *P. multocida* pathogenesis, virulence mechanisms, host specificity, and evolutionary relationships [16,17]. Since the first complete *P. multocida* genome (strain Pm70) was sequenced in 2001, over 200 genome sequences have become available, enabling comprehensive comparative analyses [18,19]. These genomic studies have identified numerous putative virulence-associated genes, including those encoding capsular polysaccharides, LPS biosynthesis factors, outer membrane proteins, adhesins, iron acquisition systems, and toxins [20,21]. Comparative genomic analyses have revealed that virulence determinants can vary significantly among strains isolated from different host species and geographic regions, and that high-virulent and low-virulent strains often differ in their repertoire of insertion sequences, prophage regions, and specific virulence genes [22,23].

Moreover, recent genomic investigations have demonstrated that *P. multocida* strains exhibit considerable genetic diversity and that certain genomic islands, mobile genetic elements, and strain-specific genes are associated with enhanced pathogenicity and host adaptation [24,25]. Studies comparing *P. multocida* isolates from respiratory infections in various livestock species have identified candidate genes potentially involved in fitness and pathogenicity, contributing to the development of more effective diagnostic tools, vaccines, and therapeutic strategies [13,26]. For instance, a WGS analysis of *P. multocida* B:2 isolates from cases of hemorrhagic septicemia (HS) in wild animals and livestock revealed clear phylogenetic clustering. The study also identified specific mutations in virulence-related genes, suggesting that gene deletions, such as flp1, might play a role in differentiating pathogenic mechanisms [27]. Furthermore, the pan-genome structure showed diversity in low-virulence factors but a more open genomic diversity pattern, offering new insights into the molecular basis of HS pathogenesis.

Another comparative genomic study from Australia, which analyzed 59 clinical isolates of *P. multocida* from pets, livestock, and captive wildlife, compared them with global reference genomes [28]. The results revealed diverse phylogenetic branches based on host origin and uncovered mobile genetic elements widely distributed across isolates. These mobile elements are linked to antibiotic resistance and potential adaptability, further highlighting the genomic flexibility of *P. multocida* across different host species.

Additionally, genomic studies of highly virulent *P. multocida* strains responsible for hemorrhagic septicemia in cattle and sheep suggest that some local high-risk strains (e.g., those from Tibetan yaks in China) accumulate genes that support metal ion homeostasis and collagen binding functions. These features likely contribute to host colonization and virulence expression, making them potential targets for future vaccine or targeted intervention strategies [29].

Despite the long-standing recognition that *P. multocida* has been extensively studied as an important veterinary pathogen, detailed genomic information on isolates derived from sheep respiratory cases in certain regions remains limited. In particular, comprehensive genomic characterization of *P. multocida* isolates associated with respiratory disease in Tan sheep from the Ningxia Hui Autonomous Region of China has been rarely reported.

The present study was therefore designed as a case-based investigation rather than a flock-level epidemiological or clinical study. A *P. multocida* isolate obtained from the lung tissue of a single Tan sheep with fatal respiratory disease was subjected to whole-genome sequencing and comparative genomic analysis. The objectives of this work were to describe the genomic features, mobile genetic elements, putative virulence-associated genes, antimicrobial resistance determinants, and phylogenetic relationships of this clinically associated isolate. By adopting a descriptive pathogenomic approach, this study aims to provide baseline genomic data for *P. multocida* circulating in Tan sheep and to contribute to future studies on respiratory disease surveillance and control in sheep.

## 2. Materials and Methods

### 2.1. Ethics Statement

All animal experiments were carried out in compliance with the guidelines of the Laboratory Animal Welfare and Ethics Committee of the Ningxia Academy of Agriculture and Forestry Sciences, ethical approval code: NXNKYKJLL-2025-12.

### 2.2. Strain Isolation and Identification

At the time of sampling, only one sheep within the flock presented with severe clinical respiratory disease. Therefore, the present study was conducted as a case-based investigation, and no systematic flock-level clinical examination or epidemiological assessment was undertaken.

To isolate the causative agent, lung tissue samples were collected aseptically from a Tan sheep in a moribund state with severe respiratory symptoms in Yanchi County, Wuzhong City, Ningxia Hui Autonomous Region, China. The samples were homogenized and centrifuged, and the resulting pellet was streaked onto Trypticase Soy Agar (TSA) plates using a sterile inoculation loop within a biosafety cabinet. The inoculated plates were incubated at 37 °C overnight. Single colonies with typical morphology were subcultured on TSA for purification, and parallel cultures were grown in Trypticase Soy Broth (TSB) at 37 °C with shaking at 200 rpm for 4–6 h. In addition, pure cultures were obtained by streaking onto Brucella Agar plates and incubating for 24 h. The representative isolate was designated P6. Under the culture conditions applied, no additional bacterial species were recovered from the lung tissue samples.

Morphological characteristics were initially assessed, and biochemical profiling was performed to support species identification. For molecular identification, bacterial genomic DNA was extracted using the TIANamp Bacterial DNA Kit (Tiangen, China). The 16S rRNA gene was amplified using primers described by Townsend et al., and the resulting sequences were compared with reference sequences in the GenBank database. Species identification of *Pasteurella multocida* was further confirmed by PCR amplification of the *P. multocida*-specific *kmt-1* gene. Moreover, capsular typing was conducted by PCR targeting serogroup-associated genes (A: *hyaD–hyaC*, B: *bcbD*, D: *dcbF*, E: *ecbJ*, F: *fcbD*) [11], which identified the isolate as capsular type A.

### 2.3. Murine Infection Model for Supportive Assessment of Virulence Potential

*P. multocida* preserved in glycerol stocks was revived in tryptic soy broth (TSB) and cultured to an optical density (OD) of approximately 0.6. The bacterial concentration was determined by serial tenfold dilution (10^1^–10^8^) according to a previously described method, and was set at 1.58 × 10^6^ colony-forming units (CFU) for challenge [30]. This specific dose was chosen because it represents a standard challenge dose in murine pasteurellosis models, which is known to produce consistent and observable clinical disease while permitting the assessment of disease progression over a relevant time frame.

To provide supportive evidence of virulence potential under controlled experimental conditions, six female, 8-week-old Institute of Cancer Research (ICR) mice obtained from the Comparative Medicine Centre of Ningxia Medical University (Yinchuan, China) were randomly allocated into two groups (*n* = 3 per group). The experimental group received an intraperitoneal injection of the *P. multocida* suspension (1.58 × 10^6^ CFU), whereas the control group was injected with sterile phosphate-buffered saline (PBS). Each group was housed separately, and food intake, general behavior, and survival were monitored throughout the observation period.

Mice that succumbed during the experiment were immediately subjected to necropsy for gross pathological examination of the lungs. At 24 h post-inoculation, surviving mice were humanely euthanized by cervical dislocation under ether anesthesia. Lung tissues exhibiting visible lesions were aseptically collected for bacterial re-isolation and identification, while remaining tissues were fixed, processed, and subjected to hematoxylin and eosin (H&E) staining. Histopathological examination was performed using a biological microscope (Olympus, Tokyo, Japan).

Given the descriptive, case-based nature of this study and the small group size (*n* = 3 per group) in this pilot assessment of virulence potential, the data derived from the mouse experiment are presented descriptively. The outcomes are qualitative and include: (1) the success of bacterial re-isolation from lung tissues, confirming the presence of viable *P. multocida*; (2) descriptive records of gross pathological lesions observed during necropsy; and (3) descriptive histopathological findings based on microscopic examination. No quantitative bacterial loads (CFU/g) were determined, and formal statistical comparisons were not performed due to the design and sample size.

### 2.4. Genome Sequencing and Annotation

The complete genomes of strain P6 were sequenced by Shanghai Sangon Biotech Co., Ltd. (Sangon, Shanghai, China) using the BGI DNBSEQ-T7 platform (BGI Group, Shenzhen, China). Raw sequencing reads were subjected to quality control using Trimmomatic v0.38 [31]. During this step, adaptor sequences were trimmed, reads containing more than 5% unidentified bases (“N”) were discarded, and sequences with over 10% of bases below a Phred quality score of 30 were removed. High-quality reads were then assembled de novo with SPAdes v3.12.0, accessed via the Pathosystems Resource Integration Center (PATRIC) online platform (https://www.patricbrc.org) [32]. Genome annotation was performed using the PATRIC comprehensive genome analysis service, which applies the Rapid Annotation Subsystem Technology (RAST) pipeline [32]. This workflow provided functional assignments for predicted coding sequences (CDS), including associations with Gene Ontology (GO) terms, classification into protein families (PLFams and PGFams), and enzyme commission (EC) numbers. Functional categorization of CDS was further refined by comparison with the COG (Clusters of Orthologous Groups) database through the WebMGA platform [33].

Pathway-level information was obtained using the KEGG Automatic Annotation Server (KAAS) (https://www.genome.jp) [32]. A Circos plot (http://www.circos.ca) was created to illustrate the genomic features and patterns of gene distribution, GC content, and GC skew.

### 2.5. Phylogenetic Analysis Based on Housekeeping Gene

To establish the phylogenetic position of *P. multocida* P6, seven housekeeping genes (*adk*, *aroA*, *deoD*, *zwf*, *gdhA*, *mdh*, and *pgi*) were extracted from the complete genome assembly and subjected to comparative analysis. NCBI BLAST (https://blast.ncbi.nlm.nih.gov/Blast.cgi, accessed on 8 September 2025) searches identified fourteen closely related strains, which were included in downstream analyses. Evolutionary relationships among these isolates were inferred through Maximum Likelihood method with bootstrap values of 1000 replicates, implemented in MEGA version 7.0.26 (http://www.megasoftware.net).

### 2.6. Mobile Genetic Elements and Bacterial Defense Systems

#### 2.6.1. CRISPR-Cas System Analysis

CRISPR arrays and associated Cas proteins were identified using CRISPRCasFinder with default parameters [34]. The identified CRISPR loci were manually curated to determine the organization of direct repeats (DR) and spacer sequences (SPA).

#### 2.6.2. Genomic Island Prediction

Genomic islands were predicted using IslandViewer 4 [35], which integrates multiple prediction methods including IslandPath-DIMOB, SIGI-HMM, and IslandPick. Predicted genomic islands were functionally annotated to identify genes associated with horizontal gene transfer, pathogenicity, and environmental adaptation.

#### 2.6.3. Prophage Element Identification

Prophage regions were identified using PHASTER [36]. PHASTER classifies prophage regions as intact, questionable, or incomplete based on completeness score, region length, and the presence of hallmark phage genes. Prophage-associated genes including integrases, terminases, capsid proteins, tail proteins, and portal proteins were systematically catalogued.

#### 2.6.4. Transposon and Insertion Sequence Analysis

Transposable elements were characterized by screening for insertion sequences through BLASTn searches against the ISFinder database (https://isfinder.biotoul.fr/) with an e-value threshold of 1 × 10^−10^ [37] and annotated based on sequence similarity to known insertion sequences (IS) and transposases.

Tandem repeats were detected using Tandem Repeats Finder v4.09 [38] with default parameters. Plasmid sequences were identified and characterized using PlasmidFinder v2.1 [39] and compared against the PlasmidFinder database.

### 2.7. Metabolic and Functional Genomics Analysis

#### 2.7.1. Secondary Metabolite Biosynthetic Gene Cluster Analysis

Biosynthetic gene clusters (BGCs) encoding secondary metabolites were predicted using antiSMASH version 6.0 (https://antismash.secondarymetabolites.org/) [40] with default parameters. The identified gene clusters were classified based on predicted product types, including nonribosomal peptides (NRPS), polyketides (PKS), terpenes, and others. Functional annotation of genes within the clusters was performed to identify biosynthetic, regulatory, and transport genes.

#### 2.7.2. Carbohydrate-Active Enzymes (CAZymes) Analysis

CAZymes were identified and classified using the dbCAN3 [41], which integrates HMMER, DIAMOND, and Hotpep tools. Predicted CAZymes were assigned to functional families including glycosyltransferases (GT), glycoside hydrolases (GH), carbohydrate esterases (CE), auxiliary activities (AA), and carbohydrate-binding modules (CBM) based on the CAZy database [42]. Sequence coverage and E-values were calculated to assess the reliability of predictions.

### 2.8. Pathogenicity and Virulence Analysis

#### 2.8.1. Virulence Factor Database (VFDB) Analysis

Virulence factors were identified by BLASTP searches against the Virulence Factors of Pathogenic Bacteria database (VFDB, http://www.mgc.ac.cn/VFs/, accessed on 8 September 2025) [43]. The analysis employed the following thresholds: minimum sequence identity of 25%, minimum coverage of 50%, and E-value cutoff of 1 × 10^−5^. Genes related to virulence and antimicrobial resistance were additionally cross-referenced with PATRIC’s specialty gene database [32].

#### 2.8.2. Pathogen–Host Interaction (PHI-Base) Analysis

Pathogenicity-related genes were identified by BLASTP searches against the Pathogen–Host Interactions database (PHI-base v4.5) [44], search parameters included: E-value threshold of 1 × 10^−5^, minimum sequence identity of 25%, and minimum query coverage of 50%. Genes were classified based on mutant phenotypes (reduced virulence, loss of pathogenicity, unaffected pathogenicity, increased virulence) and functional categories as annotated in PHI-base.

#### 2.8.3. Antimicrobial Resistance Gene Analysis

Predictions of antibiotic resistance determinants were performed using the Comprehensive Antibiotic Resistance Database (CARD, https://card.mcmaster.ca/) integrated within the PATRIC annotation framework [45]. Resistance genes were classified according to drug class (beta-lactam, aminoglycoside, fluoroquinolone, macrolide, tetracycline, sulfonamide, etc.) and resistance mechanism (antibiotic efflux, antibiotic inactivation, antibiotic target alteration, antibiotic target protection, etc.).

#### 2.8.4. Secretome and Signal Peptide Prediction

The secretome of strain P6 was characterized using SignalP version 5.0 (https://services.healthtech.dtu.dk/service.php?SignalP-5.0, accessed on 8 September 2025) to identify proteins containing N-terminal signal peptides [46]. The analysis was conducted using the Gram-negative bacteria model with the following output parameters: discrimination score (D-score) threshold of 0.5, signal peptide probability, and classification into Sec/SPI (standard secretory pathway), Sec/SPII (lipoprotein signal peptides), Tat/SPI (twin-arginine translocation pathway), or cytoplasmic proteins. Proteins were categorized as SignalP-noTM (classical secreted proteins without transmembrane domains) or SignalP-TM (signal peptide with transmembrane domain, likely membrane-anchored).

#### 2.8.5. Protein Secretion System Analysis

Bacterial secretion systems were identified using the TXSScan web tool implemented in the Galaxy/MacSyFinder environment (https://galaxy.pasteur.fr) [47]. The search encompassed all major secretion system types, including Type I through Type VI secretion systems (T1SS–T6SS), Sec-SRP (general secretory pathway), and Tat (twin-arginine translocation) pathways. Functional annotation of secretion system components was further validated through KEGG pathway database mapping and manual literature curation. Complete, partial, and remnant secretion systems were distinguished based on the presence of essential core components.

## 3. Results

### 3.1. Mouse Lethality Assay

To investigate the virulence of *P. multocida* strain P6, mice were inoculated with an infectious dose of 1.58 × 10^6^ CFU. Twenty hours post-inoculation, distinct physiological changes were observed between the experimental and control groups. Mice in the control group maintained normal appetite, while those in the experimental group exhibited symptoms such as disheveled fur, lethargy, and cold intolerance.

At 24 h post-inoculation, necropsy of the infected mice revealed significant pathological changes in the lungs. The lungs exhibited congestion, edema, and pulmonary adhesions, indicating severe pulmonary injury. Histopathological examination of lung tissue showed enlarged alveolar septa, intra-alveolar hemorrhages, and fibrinous exudation, further confirming the extent of lung damage.

**Figure 1:**
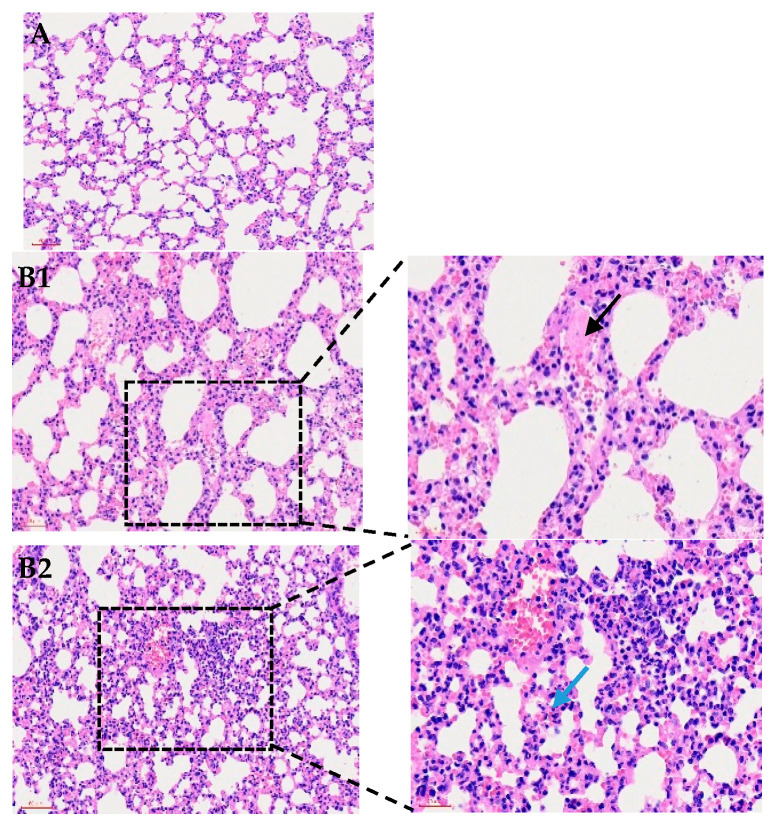

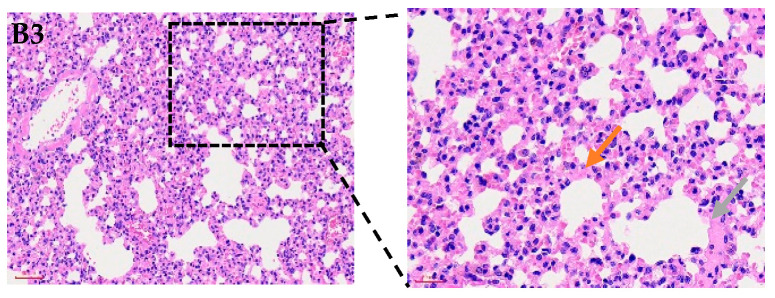
Histological examination of mouse lungs (200×) following infection with P. multocida strain P6. (**A**) shows normal lung morphology. (**B1**) presents alveoli filled with numerous neutrophils and pink proteinaceous exudate (black arrow). (**B2**) displays thickened alveolar walls with heavy interstitial inflammatory infiltration and congested capillaries (blue arrow). (**B3**) highlights fibrinous exudation and inflammatory cell accumulation (orange arrow), as well as consolidation with blurred alveolar–interstitial boundaries indicative of severe tissue injury (gray arrow).

Bacterial identification via PCR confirmed the presence of *P. multocida* P6 in all infected mice, as evidenced by consistent electrophoresis band patterns, which validated successful infection (Figure 1).

### 3.2. Genome Sequencing of P. multocida Strain P6

The strain P6 exhibits a range of notable genomic features. Its genome size is 2,289,251 bp, which is 31,764 bp larger than the avian origin *P. multocida* serogroup F strain of Pm70. P6 with a GC content of 40.2%. The genome contains 2155 coding genes, with a total gene length of 2,055,522 bp and an average gene length of 953.84 bp. Additionally, the intergenic regions span 233,729 bp, representing 10.21% of the total genome, with a GC content of 33.76%.

**Figure 2:**
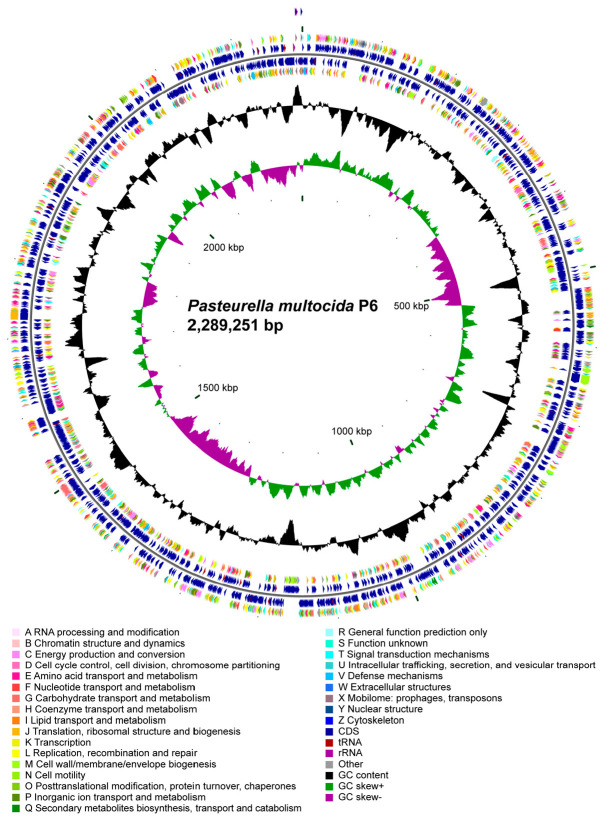
Circular genome map of *Pasteurella multocida* P6 (2,289,251 bp). The circular map displays the genomic architecture of *P. multocida* P6. From the outer to the inner rings: forward-strand coding sequences (CDSs) are color-coded according to COG functional categories, followed by reverse-strand CDSs annotated with the same scheme. Additional rings indicate the positions of tRNA and rRNA genes, as well as other annotated genomic features. The innermost plots show GC content (black) and GC skew [(G − C)/(G + C)] with positive values in green and negative values in purple, revealing local variations associated with replication origin and terminus. The map summarizes functional gene distribution, genomic composition, and structural characteristics of the P6 chromosome.

The genome is also enriched with various genetic elements, including tandem repeats ranging from 6 to 324 bp, contributing 0.42% of the genome length. There are 44 tandem repeats in total, and the genome harbors 3 genomic islands, with a combined length of 116,681 bp. Furthermore, the strain P6 contains 2 prophages, totaling 63,029 bp, one of them was intact and the other was incomplete, along with 1 CRISPR sequences and 49 tRNA genes. Additionally, the genome includes 14 plasmids, 5 rRNA genes, and 62 sRNA genes. The schematic circular map of P6 genome is listed in Figure 2.

### 3.3. Phylogenetic Analysis of P. multocida Strain P6

**Figure 3:**
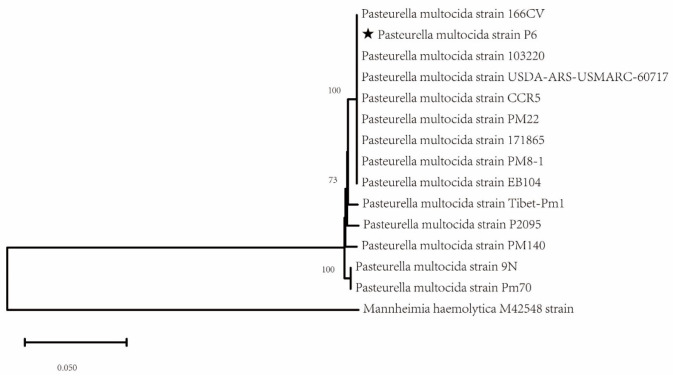
Shows the housekeeping gene phylogenetic tree of *Pasteurella multocida* strain P6. This phylogenetic tree was constructed using the Maximum Likelihood method, with the P6 isolate marked with a ★. Mannheimia haemolytica M42548 strain used as an outgroup.

To better understand the genetic relationships of conserved genes in the *P. multocida* strain P6, a phylogenetic tree was constructed, incorporating additional *P. multocida* strains isolated from various regions and hosts (Table S1). The analysis revealed that the strain P6 is genetically closest to the *P. multocida* strains 166CV and 103220, with both strains clustering together on the same branch. In contrast, significant genetic divergence was observed between strain P6 and strains 9N (rodent) and PM70 (avian) (Figure 3). This phylogenetic analysis offers valuable insights into the evolutionary relationships and genetic connections of the *P. multocida* strain P6, emphasizing its distinct lineage within the broader diversity of *P. multocida* strains.

### 3.4. Mobile Genetic Elements Analysis

#### 3.4.1. CRISPR-Cas System Organization

CRISPR-CasFinder analysis identified multiple CRISPR loci comprising 60 repeat–spacer units in the genome of strain P6 (Table S2). The CRISPR arrays, composed of 28-bp direct repeats (DRs) and 32-bp spacers (SPAs), exhibited a typical architecture; most were located on major scaffolds and bounded by conserved cas genes, suggesting the presence of functional CRISPR-Cas systems.

The consensus repeat motifs exhibited high conservation within individual loci but varied across different arrays, suggesting origins from distinct mobile genetic elements. The presence of multiple CRISPR arrays underscores the dynamic evolutionary history of strain P6 and its robust adaptive immune capability against foreign genetic elements, such as phages and plasmids.

#### 3.4.2. Genomic Island Composition

A single genomic island (designated GI01) was identified in the genome of strain P6 on Scaffold20. This ~25.4 kb region was populated with genes associated with mobile genetic elements, including an integrase and several phage-related proteins (e.g., phage tail protein, phage antirepressor). Additionally, genes encoding proteins of unknown function were present, including a DUF6246 family protein and a TM2 domain-containing protein. The prevalence of mobile element-related genes strongly suggests that GI01 was acquired through horizontal gene transfer (Table S3).

#### 3.4.3. Prophage Regions

Prophage prediction in strain P6 revealed several genomic regions of phage origin. Detailed annotation of two such regions, Ph01 and Ph02 (Table S4), confirmed the presence of genes essential for phage lytic–lysogenic cycles, such as structural, packaging, and regulatory proteins. A key distinction was observed: Ph01 on Scaffold17 encodes a cohesive set of morphogenesis genes, indicative of an intact, potentially functional prophage. In contrast, Ph02 on Scaffold20 is a mosaic region, carrying phage genes alongside non-phage elements like a toxin–antitoxin system, suggesting it is a degenerated relic. This mosaic nature highlights the role of prophages as vectors for horizontal gene transfer and drivers of bacterial genome evolution.

#### 3.4.4. Transposon Elements

Two distinct transposon regions were identified in the genome of strain P6 (Table S5). These included one element from the LINE family and one from the ISC1316 family, with lengths of approximately 0.4 kb and 1.1 kb, respectively. The high-confidence matches (E-values: 2.00 × 10^−12^ and 1.00 × 10^−114^) indicate strong sequence similarity to known mobile genetic elements. Their presence, albeit limited in number, underscores the potential for genomic plasticity in strain P6.

Together, the coexistence of CRISPR-Cas systems, genomic islands, prophages, and transposons demonstrates that strain P6 harbors a highly dynamic and mosaic genome. A direct comparison with the reference strain *P. multocida* PM70 clarifies its specific mobile genetic element (MGE) profile: while PM70 possesses two genomic islands and four CRISPR arrays, strain P6 carries one genomic island and two CRISPR arrays. Conversely, P6 encodes two transposon elements, compared to one in PM70. This quantitatively intermediate and distinct profile suggests that strain P6 maintains a balanced genomic architecture—it sustains substantial defensive and adaptive capabilities through its MGEs, yet does not exhibit an extreme accumulation of any single type. These mobile elements collectively contribute to genetic variability, environmental adaptability, and defense against foreign DNA. The combination of defense (CRISPR-Cas) and mobility (islands, prophages, transposons) underpins an intricate equilibrium between genome stability and plasticity, which is consistent with the evolutionary strategy of environmental Pasteurella species thriving in diverse niches.

### 3.5. Metabolic System Analysis

#### 3.5.1. Secondary Metabolite Biosynthetic Gene Cluster Analysis

##### Gene Cluster Organization

AntiSMASH analysis of strain P6 identified several candidate secondary metabolite biosynthetic gene clusters. Among them, a principal cluster (cluster 1) spanning approximately 32 kb was located on a major scaffold (Scaffold 1). The organization of cluster 1 is linear, with genes grouped into biosynthetic, regulatory, and transport modules. Each functional category was clearly delineated during analysis using distinct annotation tracks.

##### Functional Gene Classification

The genes within cluster 1 were classified into the following functional categories:

(i) Biosynthetic genes. These include core and auxiliary biosynthetic genes. The core biosynthetic genes comprise ycaO (encoding a 30S ribosomal protein S12 methylthiotransferase accessory factor) and a class I SAM-dependent methyltransferase. Key auxiliary biosynthetic genes include ubiX (flavin prenyltransferase), moeB (molybdopterin-synthase adenylyltransferase), and acs (acetate–CoA ligase), which are predicted to supply cofactors or generate key biochemical intermediates necessary for metabolite assembly.
(ii) Regulatory genes. This category includes specific transcriptional regulators, notably an FNR-family transcription factor, suggesting that cluster expression may be regulated in response to environmental conditions.
(iii) Transporter genes. These genes encode components of a peptide ABC transporter system, consistent with a role in transmembrane trafficking of pathway intermediates or the final metabolic product.
(iv) Auxiliary biosynthetic genes. Designated as the biosynthetic-additional category, these genes encode supportive enzymatic functions, such as a CPBP family glutamic endopeptidase and a hydroxyacylglutathione hydrolase, which may assist in core biosynthetic steps.
(v) Other genes. This group includes genes with housekeeping, basal metabolic, or unknown functions (e.g., xthA, sppA, folE) that are located within or adjacent to the cluster. These may contribute to the local genomic and transcriptional environment (Table S6).

#### 3.5.2. Carbohydrate-Active Enzymes Analysis of Strain P6

##### Overall CAZyme Repertoire

The genome encodes 60 CAZymes distributed across five classes (Table S7): glycosyltransferases (GT), 33 (55.0%); glycoside hydrolases (GH), 14 (23.3%); carbohydrate esterases (CE), 9 (15.0%); auxiliary activities (AA), 3 (5.0%); and carbohydrate-binding modules (CBM), 1 (1.7%). This GT-dominant profile is consistent with robust capsular polysaccharide biosynthesis and lipooligosaccharide modification, which are critical for the virulence and host adaptation of Pasteurella species.

##### GT Families

GT genes span multiple families, with enriched representation in GT4 (3 genes: gene1049, gene1054, gene1557; coverage 36–39%; E = 1.6 × 10^−9^–7.5 × 10^−36^), GT9 (5 genes: gene0900, gene0901, gene1324, gene1335, gene1551; coverage 65–69%; E = 1.8 × 10^−39^–4.8 × 10^−55^), GT25 (3 genes), and GT41 (4 genes).

Notably, sialylation-related GTs were detected, including GT52 (gene0431), GT80 (gene0702), and GT42 (gene1525), with high coverage (~83–96%) and the highly significant E value (~1.2 × 10^−88^–1.1 × 10^−161^) suggests that P6 has the potential for acidic polysaccharide/capsule and immune escape related glycosylation.

##### GH Families

We identified 14 GH genes across multiple GH families, led by GH13 subfamilies (3 genes: gene0395, gene0396, gene0504; coverage 41–62%; E = 1.0 × 10^−152^–3.0 × 10^−173^), involved in α-glucan metabolism. gene0397 (GH77) encodes a 4-α-glucanotransferase with 78% coverage and E = 4.2 × 10^−143^. Two GH33 sialidases (gene0273, gene1060; coverage 44–45%; E = 4.0 × 10^−64^ and 2.2 × 10^−102^) suggest capacity for host glycoconjugate modification.

Peptidoglycan-targeting hydrolases include GH23 (gene0548, gene1305) and GH103 (gene0426; coverage 87%; E = 7.2 × 10^−99^), consistent with cell-wall remodeling and potential roles in growth or niche adaptation.

##### CE Families and CBM

Nine CE genes were assigned to CE1/CE3/CE9/CE11/CE16. CE1 predominated (5 genes: gene0052, gene1219, gene1586, gene1587, gene1917; coverage 36–91%; gene1587 with 91%, E = 3.1 × 10^−22^), consistent with acetylxylan/feruloyl esterase-like functions and cell-envelope tailoring. CE9 (gene0044; 98%; E = 6.3 × 10^−112^) and CE11 (gene0662; 90%; E = 6.1 × 10^−122^) likely mediate peptidoglycan precursor deacetylation essential for cell-wall integrity. A single CBM50 (gene1942; 33%; E = 7.1 × 10^−15^) indicates peptidoglycan/chitin-binding potential frequently associated with GH cell-wall enzymes.

##### Synthesis

The NRPS/PKS-rich BGC landscape and GT/GH/CE-centered CAZy toolkit together suggest a metabolically adaptable chassis in strain P6, capable of surface glycan remodeling, capsule/LPS diversification, and secretion/efflux of secondary metabolites—features that may enhance biofilm formation, intermicrobial competition, and host/environmental interactions.

### 3.6. Pathogenic System Analysis

#### 3.6.1. Identification of Pathogenicity-Related Genes

BLASTP searches against the PHI-base identified 647 putative pathogenicity-related genes (Table S8), distributed across 37 scaffolds, with the highest density observed on Scaffold1 (152 genes, 23.5%). Sequence identity to known virulence factors averaged 48.3% (median: 45.5%, range: 25.6–89.2%) with an average coverage of 87.5% (median: 96.4%). Using stringent criteria (identity > 50%, coverage > 80%), 238 genes (36.8%) were classified as high-confidence orthologs. The most frequent pathogen homologs were derived from Escherichia coli (76 genes), Salmonella enterica (70 genes), and Pasteurella aeruginosa (43 genes).

These findings indicate that strain P6 shares conserved virulence determinants with both enteric and opportunistic pathogens, highlighting its potential to express broad-spectrum pathogenic capabilities.

#### 3.6.2. Phenotypic Classification of Virulence Genes

Based on experimentally validated mutant phenotypes in PHI-base, the genes were grouped into distinct categories (Table S9). The majority exhibited reduced virulence phenotypes (378 genes, 58.4%), indicating their requirement for full pathogenicity. A substantial fraction (123 genes, 19.0%) showed unaffected pathogenicity, suggesting possible redundancy or compensatory functions. Notably, 57 genes (8.8%) were associated with increased virulence (hypervirulence) phenotypes, implying potential roles as negative regulators of pathogenicity. Additionally, 22 genes (3.4%) were classified as essential, with mutations causing a complete loss of pathogenicity.

The predominance of reduced virulence phenotypes suggests that strain P6 relies on a complex network of nonredundant virulence mechanisms to maintain full infectivity.

#### 3.6.3. Functional Classification of Pathogenicity Genes

Functional annotation inferred from gene descriptions and homology-based classification revealed diverse roles among the virulence-associated loci (Table S10). Metabolic enzymes represented the largest class (174 genes, 26.9%), including pathways for amino acid biosynthesis (aroA, trpB, leuC), nucleotide metabolism (guaB, carA), and central carbon metabolism (galE, aceF). Transport systems accounted for 108 genes (16.7%), dominated by ABC transporters and permeases. Regulatory genes (58 genes, 9.0%) encompassed global regulators such as crp, fnr, and arcA, as well as RNA chaperones (hfq) and stringent response modulators (dksA). Additional categories included DNA/RNA metabolism (30 genes, 4.6%), secretion-related components (19 genes, 2.9%), cell envelope biosynthesis (13 genes, 2.0%), and adhesion factors (6 genes, 0.9%).

These results indicate that the pathogenicity potential of strain P6 arises from an intricate interplay between metabolism, regulation, and secretion processes.

#### 3.6.4. Characterization of the Secretome

SignalP 5.0 analysis identified 142 proteins containing N-terminal signal peptides (Table S11). These were predominantly distributed across Scaffold1 (46 proteins, 32.2%) and Scaffold2 (36 proteins, 25.2%). D-scores ranged from 0.517 to 0.953 (mean: 0.735 ± 0.093), with signal peptide probability scores averaging 0.913 ± 0.057. Classification indicated that 141 proteins (99.3%) were of the SignalP-noTM type, suggesting classical Sec or Tat pathway-mediated secretion, whereas only two proteins (1.4%) were SignalP-TM, likely membrane-anchored forms. Fifteen proteins exhibited high-confidence secretion signals (D-score > 0.90), including gene0462 (0.953), gene1409 (0.930), and gene0691 (0.927).

These predicted secreted proteins represent a substantial extracellular proteome that may mediate host interaction, environmental sensing, and nutrient uptake.

#### 3.6.5. Protein Secretion System Architecture

Systematic annotation revealed the presence of complete Sec-SRP and Tat secretion pathways (Table S12). The Sec-SRP system comprised all essential components, including the SecYEG translocon (secY, secE, secG), motor ATPase SecA (secA), accessory SecDF–YajC complex (secD, secF, yajC), chaperone SecB (secB), and SRP elements Ffh (ffh) and FtsY (ftsY). The YidC insertase (yidC) was also identified, supporting membrane protein insertion. The Tat system was intact, containing tatA, tatB, and tatC, enabling the translocation of folded proteins across the inner membrane. Only a single gene corresponding to the Type VI secretion system kinase PpkA (ppkA) was detected, with other T6SS components absent.

Collectively, these data indicate that strain P6 maintains a conserved general secretion framework, complemented by a partial T6SS module potentially involved in interbacterial interactions.

#### 3.6.6. High-Confidence Core Virulence Determinants

Applying stringent criteria (identity > 60%, coverage > 90%, and reduced virulence phenotype) identified 108 high-confidence virulence genes (Table S13). Top conserved determinants included pdxS (89.2%) and pdxT (62.3%) involved in pyridoxine biosynthesis, tnaA (89.0%) encoding tryptophanase, iscU (86.7%) for Fe–S cluster assembly, and galE (86.3%) encoding UDP-glucose epimerase. Key regulatory genes such as crp (85.9%), hfq (82.8%), and dksA (82.4%) also showed strong conservation, underscoring their roles in global virulence regulation.

These conserved determinants correspond to central virulence networks commonly observed in Pasteurella species, suggesting evolutionary conservation of regulatory and metabolic mechanisms underlying pathogenicity.

### 3.7. Comparative Analysis

#### 3.7.1. Comparative Analysis of Virulence Factors

Comparative profiling against the Virulence Factor Database (VFDB) identified 187 virulence-associated genes shared between *P. multocida* P6 and the reference strain PM70 (AE004439) (Table S14). This conserved virulence repertoire primarily encompassed genes related to capsular biosynthesis, lipooligosaccharide (LOS) assembly, adherence (e.g., Flp pili, filamentous hemagglutinin), iron acquisition systems (e.g., HitABC, Chu), and various secretion system components.

Among these, 114 genes (60.9%) were fully conserved, constituting a core virulome for the species. These included key determinants such as capsular polysaccharide synthesis enzymes (e.g., Cap8F, BexD), outer membrane proteins (e.g., OmpP2, OmpA), and heme/hemoglobin utilization proteins (e.g., ChuU, HmbR).

Notably, strain P6 exhibited variations in gene copy number for specific virulence-associated factors compared to AE004439. For instance, P6 possessed fewer copies of a hemoglobin-binding protein (Hgp) but had more copies of elongation factor Tu (EF-Tu)—a moonlighting protein involved in adhesion—and genes related to a direct heme uptake system. Furthermore, P6 uniquely encoded a putative TonB-dependent outer membrane receptor for transferrin and lactoferrin-mediated iron uptake, which was absent in the reference strain.

These findings indicate that while *P. multocida* P6 retains the fundamental virulence backbone of the species, specific variations and acquisitions in its virulome may reflect fine-tuning for adaptation to particular host niches.

#### 3.7.2. Comparative Analysis of Antimicrobial Resistance Genes

Comparative screening against the Comprehensive Antibiotic Resistance Database (CARD) identified 65 antibiotic resistance genes (ARGs) across 10 drug classes in the analyzed strains (Table S15). Both *P. multocida* P6 and PM70 harbored a highly similar and diverse profile of intrinsic resistance determinants, dominated by genes encoding efflux pumps from the ABC transporter superfamily (e.g., macB, msbA) and enzymes mediating antibiotic target alteration (e.g., eptA, Erm (31)) or target protection (e.g., various tet genes).

While the overall ARG profiles were congruent, key differences were observed. Strain P6 acquired lsaB, an ABC-F protein gene conferring potential resistance to lincosamides, but lost the ribosomal protection protein genes tet(M) and vmlR, as well as the regulator adeL, which were present in PM70. A significant finding was the presence of core components of the vanA gene cluster (e.g., vanH, vanR) in both strains, which is uncommon in Pasteurella and strongly suggests historical horizontal gene transfer events.

Overall, both *P. multocida* strains possess a rich and complex resistome largely composed of intrinsic resistance genes. P6 does not exhibit a broader resistance spectrum than PM70; rather, the differences lie in the specific assortment of acquired and intrinsic genes, potentially indicative of distinct evolutionary paths under different selective pressures.

#### 3.7.3. Comparative Analysis of COG

We compared the COG functional profiles of the Pasteurella multocida local isolate P6 and the reference strain PM70 (AE004439). Overall, both strains showed highly similar distributions across major COG categories, including Information Storage and Processing and Cellular Processes and Signaling, where most functions differed only slightly in gene counts (e.g., COG J: 219 vs. 223; COG K: 99 vs. 104; COG M: 190 vs. 192). Consistently, the proportion-based statistical comparison showed no significant differences for these categories, with corrected *p* values (FDR) equal to 1.0 for all functions except one, indicating that the majority of core biological processes are conserved between the two strains (Table S16).

However, a pronounced difference was observed in COG X, which is associated with cellular processes and potential stress or environmental response functions. P6 showed a substantially higher count (29) compared with PM70 (AE004439) (5), representing the most notable functional divergence. This difference was also strongly supported by the statistical test: COG X was the only category exhibiting a significant enrichment in P6, with a corrected *p*-value of 0.000919, confirming that the expansion of COG X is not due to random variation. In addition, several metabolic categories showed moderate but non-significant increases in P6, such as COG G (carbohydrate transport/metabolism; 197 vs. 184) and COG F (nucleotide metabolism; 72 vs. 68), suggesting possible enhancement of metabolic versatility, although these differences did not reach statistical significance after correction (all FDR = 1.0) (Table S17).

Together, these results demonstrate that although P6 and AE004439 share a largely conserved functional architecture across most COG categories, the local isolate P6 exhibits a significant enrichment specifically in COG X, which may reflect adaptation to local environmental pressures or enhanced stress response capabilities. This unique expansion of COG X in P6 may contribute to strain-specific ecological fitness or pathogenic properties, warranting further experimental validation.

#### 3.7.4. Comparative Analysis of KEGG Functions

KEGG-based functional annotation revealed that the overall genomic functional profiles of strain P6 and the reference strain PM70 (AE004439) were highly conserved across all hierarchical levels. At the Level 1 classification, both strains exhibited similar distributions of major functional categories, with P6 showing slightly higher gene counts in Metabolism and Environmental Information Processing, suggesting a modest expansion in genes associated with metabolic activity and environmental signal processing (Table S18).

At Level 2, differences were predominantly observed within metabolic subcategories. Gene counts related to Carbohydrate metabolism, Amino acid metabolism, and Energy metabolism were marginally higher in P6 compared with AE004439, while other categories remained comparable between the two genomes. These results indicate a minor increase in metabolic pathway representation in P6 without altering the overall functional architecture.

A more detailed comparison at Level 3 demonstrated that the two strains possessed identical gene numbers in core metabolic pathways such as Glycolysis/Gluconeogenesis and the TCA cycle. However, P6 carried slightly more genes in specific carbohydrate-related pathways, including the Pentose phosphate pathway and Pentose and glucuronate interconversions. These differences were quantitative rather than structural, reflecting a subtle enrichment of certain metabolic routes in P6.

At the KO level, a total of 1641 KEGG Orthologs were identified. Comparative analysis showed that the proportional abundances of all KO terms were nearly identical between the two strains. Statistical testing revealed no significant differences, with all KO terms exhibiting *p*-values and FDR-adjusted *p*-values equal to 1, indicating complete conservation at the functional gene unit level.

Taken together, these results demonstrate that P6 and AE004439 share a highly conserved functional repertoire across all KEGG hierarchical layers. The only detectable differences were limited to slight increases in the number of genes assigned to several metabolic pathways in P6, while the absence of KO-level divergence confirms that the fundamental functional framework of both strains remains essentially unchanged.

## 4. Discussion

The present study provides a descriptive genomic overview of *P. multocida* strain P6, a clinically associated isolate recovered from the lung tissue of a Tan sheep that succumbed to severe respiratory disease. The genome size (2,289,251 bp) and GC content (40.2%) are comparable to those reported for *P. multocida* isolates from diverse hosts [13,15]. Comparative analyses indicate that P6 shares a conserved genomic backbone with other *P. multocida* strains while carrying accessory elements and mobile genetic components that may contribute to genomic plasticity and adaptation.

### 4.1. Mobile Genetic Elements and Genome Plasticity

The genome of strain P6 harbors multiple classes of mobile genetic elements (MGEs)—CRISPR–Cas loci, a genomic island, prophage regions, and transposons—indicating a dynamic, mosaic architecture shaped by recurrent horizontal gene transfer and phage predation [48,49,50]. This genomic plasticity is characteristic of environmentally adaptable Pasteurella species that frequently interact with diverse microbial communities and their mobile gene pools.

CRISPR–CasFinder analysis identified multiple CRISPR loci comprising 60 repeat–spacer units in strain P6, with arrays of conserved 28-bp direct repeats and heterogeneous 32-bp spacers. The presence of several arrays with conserved repeats but diverse spacers suggests that strain P6 has faced repeated exposure to a broad spectrum of MGEs throughout its evolutionary history, a trait common in bacterial pathogens from phage-rich environments [51]. Although no CRISPR–Cas-bearing genomic islands or plasmids were detected in P6, the modular organization of its CRISPR loci is consistent with a potentially mobile origin, as such systems are often horizontally disseminated [52,53].

The coexistence of CRISPR arrays and prophage regions suggests an interplay between defense and lysogeny. This coexistence is consistent with a balance between CRISPR-mediated defense and prophage carriage observed in many bacteria [54]; however, specific anti-defense mechanisms were not investigated in this study. Furthermore, theoretical models suggest that spacer–target mismatches can mitigate the fitness costs of self-targeting in lysogens carrying transcriptionally active prophages, a trade-off that may explain the persistence of these systems in polylysogenic populations [55]. Although the CRISPR–Cas subtypes in P6 were not resolved, these principles highlight a likely balance in P6 between maintaining broad antiviral immunity and limiting the costs associated with resident prophages.

Analysis revealed a single ~25.4 kb genomic island (GI01) on Scaffold20 in strain P6 (Section 3.4.2). GI01 is enriched in hallmark signatures of horizontal acquisition, including an integrase and several phage-related proteins (e.g., phage tail protein, antirepressor), alongside hypothetical proteins. The gene content, dominated by phage-derived components, suggests that GI01 primarily represents a phage-derived cargo island—a potential platform for accumulating fitness-associated genes—rather than a classical pathogenicity island [50,56]. In *P. multocida*, genomic islands are central to strain-specific adaptation, accounting for a significant proportion of the accessory and unique genes [57]. The presence of only one island in P6 places it at one end of the species’ spectrum, yet GI01 remains a key contributor to its accessory genome, functioning as a “fitness island” that may enhance survival in specific niches [56,58].

Prophage prediction identified several phage-derived regions, with two (Ph01 and Ph02) characterized in detail. Ph01 on Scaffold17 encodes a cohesive set of morphogenesis and regulatory proteins, consistent with an intact, potentially functional prophage. In contrast, Ph02 on Scaffold20 exhibits a mosaic organization, combining phage genes with non-phage elements like a toxin–antitoxin module, indicating a degenerated prophage relic. The co-localization of GI01 and the mosaic prophage Ph02 on Scaffold20 indicates that this region functions as a micro-hotspot of horizontal gene flux and defence gene accumulation in P6 [59,60]. Although no canonical integrative and conjugative elements (ICEs) were identified in P6, this island–prophage cluster suggests the chromosome possesses integration sites that could facilitate the future acquisition of MGEs carrying traits like antimicrobial resistance [28,61,62].

Two distinct transposon regions were detected in strain P6: a LINE family element (~0.4 kb) and an ISC1316 family element (~1.1 kb). Despite their limited number, transposons are potent drivers of local genome plasticity. They can disrupt genes, remodel regulatory regions, and mobilize adjacent DNA, thereby accelerating the diversification of accessory gene content and influencing the expression of virulence or stress-response factors [44]. In P6, the proximity of these transposons to phage-derived regions on Scaffold20 raises the possibility that transposition has contributed to the mosaic architecture of Ph02 and to the assembly or rearrangement of GI01.

Taken together, the coexistence of multiple CRISPR arrays, a phage-derived genomic island, both intact and degenerated prophages, and discrete transposon elements demonstrates that strain P6 carries a compact but highly dynamic mobilome. This arrangement reflects a delicate balance between genome stability—maintained through immune defence (CRISPR-Cas) and prophage-regulated protection—and genome plasticity, which is mediated by islands, prophages, and transposons to permit rapid adaptation [50,56,57,58,63]. Such a balance is consistent with the ecological profile of environmental Pasteurella species capable of persisting and competing across diverse niches.

### 4.2. Virulence-Associated Gene Content and Putative Pathogenic Features

The comprehensive virulence factor analysis identified 647 putative pathogenicity-related genes, with 238 classified as high-confidence orthologs based on stringent criteria. The predominance of genes exhibiting reduced virulence phenotypes (58.4%) indicates that strain P6 appears to rely on a complex, nonredundant network of virulence-associated functions that may support infection-related fitness. This finding is consistent with transcriptomic studies comparing high- and low-virulence *P. multocida* strains, which revealed extensive differential expression of virulence-associated genes [64]. The major virulence factors identified in *P. multocida* include capsular polysaccharides, lipopolysaccharides, outer membrane proteins, and iron acquisition systems [7,65].

The functional classification revealed that metabolic enzymes represented the largest class (26.9%), emphasizing the critical role of metabolic adaptation in pathogenesis. Key metabolic pathways including amino acid biosynthesis (aroA, trpB, leuC), nucleotide metabolism (guaB, carA), and central carbon metabolism (galE, aceF) have been shown to be essential for bacterial survival and virulence in various pathogens [66]. Notably, the identification of high-confidence virulence genes such as pdxS and pdxT (pyridoxine biosynthesis), tnaA (tryptophanase), and iscU (Fe-S cluster assembly) highlights the importance of nutritional competence and iron acquisition in bacterial pathogenicity.

Recent pathogenomic analyses of *P. multocida* strains recovered from human infections have provided additional insights into the role of metabolic versatility in virulence. These studies revealed that human isolates, predominantly belonging to subspecies septica, display a reduced repertoire of iron receptors compared to animal isolates, suggesting host-specific adaptation of iron acquisition strategies [26]. Furthermore, gene-trait analysis identified a putative L-fucose uptake and utilization pathway over-represented in subsp. septica strains, potentially representing a novel host predilection mechanism [26]. The presence of multiple iron acquisition systems in P6, including HitABC, Chu, and the unique TonB-dependent receptors for transferrin and lactoferrin identified in this study, indicates broad iron scavenging capabilities that may be relevant to survival across different host niches and during infection.

The detection of 142 secreted proteins with signal peptides suggests a substantial extracellular proteome involved in host interaction and environmental sensing. The complete Sec-SRP and Tat secretion pathways identified in strain P6 are consistent with the conserved general secretion framework observed in *P. multocida* and related pathogens [67,68]. The presence of a partial Type VI secretion system component (ppkA) suggests potential involvement in interbacterial competition, although the absence of other T6SS components indicates that this system may be incomplete or under evolutionary transition [68].

This genomic profile suggests a potential adaptive trade-off. The comparatively smaller set of virulence genes in strain P6 versus some reference strains may reflect niche-specific optimization rather than diminished pathogenic potential. As a facultative pathogen, P6 could have undergone genomic streamlining in a stable environment (e.g., the ovine respiratory tract), retaining core virulence machinery for efficient infection while shedding redundant factors. This adaptation aligns with its ability to cause severe disease despite a more focused virulome.

### 4.3. Carbohydrate Metabolism and Surface Glycan Remodeling

The comprehensive CAZyme analysis revealed 60 carbohydrate-active enzymes with a glycosyltransferase-dominant profile (55%), consistent with extensive cell surface and lipopolysaccharide remodeling capabilities. The identification of sialylation-related glycosyltransferases (GT52, GT80, GT42) with highly significant E-values suggests that strain P6 possesses the capacity for acidic polysaccharide synthesis and capsule formation, which are critical virulence factors in *P. multocida* pathogenesis [69].

The glycosyltransferase repertoire of P6 aligns with findings from other well-characterized *P. multocida* strains, which typically encode 33-34 GT genes primarily involved in capsule biosynthesis and lipooligosaccharide modification [21]. Recent structural and functional studies have demonstrated that specific glycosyltransferases within the capsule biosynthesis locus are essential for virulence. For instance, mutations in hyaD (encoding the capsule-specific glycosyltransferase in serotype A strains) result in acapsular phenotypes with dramatically attenuated virulence, rendering strains unable to cause systemic disease in animal models [70,71]. Moreover, genome-wide TraDIS screening identified the stringent response regulator SpoT as a negative regulator of hyaluronic acid capsule production, acting by modulating expression of capsule-specific glycosyltransferase genes such as hyaD [72]. This regulatory mechanism allows *P. multocida* to modulate capsule production in response to environmental stress, potentially facilitating adaptation to different stages of infection.

Capsular polysaccharides play essential roles in immune evasion by preventing complement-mediated killing and phagocytosis [71]. The presence of two GH33 sialidases (neuraminidases) is particularly noteworthy, as these enzymes are involved in the degradation of host glycoconjugates and may contribute to bacterial colonization and nutrient acquisition [73]. Previous studies have shown that sialic acid metabolism genes, including nanB (sialidase), are highly conserved among *P. multocida* isolates and play important roles in host adaptation [74]. The combination of sialylation and desialylation capabilities suggests sophisticated mechanisms for modulating cell surface properties and host–pathogen interactions.

Beyond capsule biosynthesis, recent investigations have unveiled extensive diversity in *P. multocida* LPS outer-core structures. Novel glycosyltransferases responsible for distinct LPS structures have been identified, including new outer-core genotypes (e.g., L9) encoding previously uncharacterized enzymes such as UDP-N-acetylglucosamine 2-epimerase homologs and mannosyltransferase-like proteins [26]. Functional studies demonstrate that LPS glycosyltransferases such as HptE and PcgD contribute differentially to virulence; while mutations in these genes produce truncated LPS with attenuated virulence, some mutants retain the ability to persist for extended periods in host tissues [75,76]. The diversity of glycosyltransferases identified in P6, spanning 16 GT families with notable enrichment in GT4, GT9, GT25, and GT41, likely contributes to structural heterogeneity in both capsule and LPS, enhancing immune evasion capabilities.

The identification of peptidoglycan-targeting hydrolases (GH23, GH103) and carbohydrate esterases involved in cell wall modification indicates active cell envelope remodeling, which is essential for bacterial growth, division, and adaptation to changing environmental conditions. The GT/GH/CE-centered CAZy toolkit, together with the secondary metabolite biosynthetic gene clusters, suggests a metabolically versatile organism capable of surface glycan diversification and biofilm formation.

### 4.4. Comparative Genomic Perspectives and Evolutionary Trajectory

Comparative analysis against VFDB identified 187 conserved virulence-associated genes shared between P6 and the reference strain Pm70, mainly related to capsule biosynthesis, lipooligosaccharide/lipopolysaccharide assembly, outer membrane proteins, adhesins, and iron acquisition systems. Overall, the conserved virulence-associated gene content suggests that P6 retains core features commonly reported for *P. multocida*, while variation in accessory genes and mobile genetic elements may reflect niche- or host-associated adaptation.

Indeed, comprehensive genomic surveys of 656 *P. multocida* isolates from diverse hosts and continents have demonstrated extensive genomic diversity with limited host or capsular serogroup specificity, with the notable exception of bovine-source isolates which form a more distinct phylogenetic cluster [61]. Whole-genome phylogeny constructed from 237,670 core SNVs revealed an overall lack of host specificity, suggesting that most *P. multocida* lineages maintain broad host range capabilities through retention of core virulence mechanisms while acquiring strain-specific adaptations through horizontal gene transfer [61]. Interestingly, outbreak investigations of fowl cholera revealed that multiple strains of *P. multocida* were circulating simultaneously within the same commercial turkey company, challenging the assumption that outbreaks are caused by single, highly virulent clones [61]. This polyclonal nature underscores the broadly distributed virulence potential across the species.

The antimicrobial resistance gene profiling identified 65 ARGs distributed across 10 drug classes, revealing that strain P6 does not exhibit a broader resistance spectrum compared to the typical *P. multocida* strain PM70. Instead, the differences lie in the specific assortment of acquired and intrinsic genes, with P6 acquiring certain genes (e.g., lsaB) but losing others (e.g., tet(M), vmlR). Notably, the presence of vanA-like and tetG genes suggests horizontal acquisition from diverse bacterial sources, potentially mediated by the extensive mobile genetic elements identified in this study [77,78]. The conservation of multidrug efflux pump systems (mexAB-oprM, mexCD-oprJ, mexEF-oprN, mexXY-oprM) alongside acquired resistance determinants indicates a multifaceted approach to antimicrobial tolerance [79]. Recent studies have emphasized the importance of monitoring antibiotic resistance in *P. multocida* from both companion animals and livestock to prevent zoonotic transmission and ensure effective therapeutic strategies [80].

The dynamic nature of the *P. multocida* resistome is further illustrated by the specific pattern of resistance gene gains and losses in strain P6. While P6 acquired lsaB (encoding an ABC-F protein conferring lincosamide resistance), it lost tet(M), vmlR, and adeL present in PM70, indicating ongoing resistome evolution through differential MGE-mediated transfer events. Recent surveillance studies have documented widespread distribution of IncQ1 plasmids and ICEs carrying multiple resistance genes across *P. multocida* populations [61]. These mobile elements frequently harbor resistance genes for aminoglycosides (18.1% of genomes), tetracyclines (17.5%), streptogramins (10.3%), macrolides (10.1%), and lincosamides (10.0%), with ICEPmu1-related elements in bovine respiratory disease isolates harboring up to 11 different antimicrobial resistance genes [61,62]. The presence of vanA gene cluster components in both P6 and PM70 is particularly unusual for Pasteurella species and strongly suggests historical horizontal gene transfer events from Gram-positive donors, highlighting the potential for acquisition of resistance mechanisms from phylogenetically distant sources.

The comparative COG functional analysis between P6 and reference strain AE004439 revealed a statistically significant enrichment in COG category X (mobilome: prophages, transposons), with P6 harboring 29 genes versus only 5 in AE004439 (corrected *p*-value = 0.000919). This pronounced difference represents the only major functional divergence between the two strains and suggests enhanced genetic plasticity in P6. The expansion of COG X may reflect adaptation to environments with high horizontal gene transfer activity and could contribute to strain-specific ecological fitness by facilitating rapid acquisition of adaptive traits. At the KEGG functional level, P6 showed modest, non-significant increases in genes related to carbohydrate metabolism pathways (particularly Pentose phosphate pathway and Pentose and glucuronate interconversions), amino acid metabolism, and energy metabolism compared to AE004439. The overall conservation of core metabolic pathways, coupled with the dramatic expansion of mobile genetic elements, supports a model of *P. multocida* evolution characterized by a stable metabolic core with a highly dynamic accessory genome.

### 4.5. Evolutionary Implications and Ecological Adaptation

The integrated analysis of mobile genetic elements, virulence factors, and antimicrobial resistance genes reveals that *P. multocida* strain P6 represents an environmentally adapted lineage that has retained core virulence-associated gene content while expanding its resistome and metabolic capabilities. The coexistence of defense mechanisms (CRISPR-Cas) and mobility elements (genomic islands, prophages, transposons) suggests an intricate balance between genome stability and adaptability, typical of bacteria thriving in diverse ecological niches [81].

Pan-genomic analyses across diverse *P. multocida* populations have confirmed this pattern, revealing an open pan-genome structure with continuous acquisition of accessory genes through horizontal gene transfer [57,82]. Analysis of over 100 strains demonstrated that accessory genes, comprising approximately 62% of the pan-genome, are significantly enriched in genomic islands and mobile genetic elements, enabling *P. multocida* to rapidly adapt to new hosts, environmental pressures, and antimicrobial challenges [82]. Furthermore, diversification selection analysis has revealed that surface-exposed proteins in *P. multocida* are subject to strong positive selection pressure, resulting in interstrain heterogeneity related to host adaptation capabilities [82]. This selection may drive the evolution of strain-specific outer membrane proteins, adhesins, and immune evasion factors that determine host range and tissue tropism.

The evolutionary trajectory of strain P6 appears to be shaped by both vertical inheritance of core pathogenic machinery and horizontal acquisition of accessory genes through mobile genetic elements. This mosaic genome architecture is consistent with the ‘quantum leap’ model of bacterial evolution, where horizontal gene transfer enables rapid acquisition of complex phenotypes [56,58]. The presence of multiple genomic islands carrying metal resistance, secretion systems, and toxin–antitoxin modules indicates that environmental selective pressures, including antimicrobial exposure and competition with other microorganisms, have played significant roles in shaping the genome of strain P6.

From a veterinary and livestock-production perspective, understanding the genomic characteristics of *P. multocida* isolates from sheep is important for improving regional disease surveillance, informing prevention strategies, and guiding rational antimicrobial use. The descriptive pathogenomic data generated for strain P6 provide a reference for future studies with expanded sampling and may support the development of locally relevant control measures, including diagnostics and vaccine-related research.

### 4.6. Study Limitations

This study has several limitations that should be acknowledged. First, the investigation was based on a single clinical case, and only one animal exhibiting severe respiratory disease was available for sampling at the time of investigation. Therefore, the findings of this study cannot be extrapolated to represent flock-level epidemiology or disease prevalence in Tan sheep.

Second, comprehensive clinical examinations of additional animals, including imaging approaches such as ultrasonography or diagnostic procedures such as bronchoalveolar lavage, were not performed. As a result, the clinical context of respiratory disease within the flock could not be fully evaluated.

Third, although a murine infection model was employed, this experiment was designed to provide preliminary, supportive evidence of virulence potential rather than to fulfill Koch’s postulates or to establish definitive causality. Additionally, the small group size (*n* = 3) in this model limits its statistical power and the generalizability of the virulence assessment. Taken together, these limitations indicate that the present work should be interpreted as a descriptive, case-based genomic characterization of a clinically associated *P. multocida* isolate.

Despite these limitations, the genomic data generated in this study provide a valuable reference for *P. multocida* isolates circulating in Tan sheep and may serve as a foundation for future epidemiological investigations and experimental studies with expanded sample sizes.

## 5. Conclusions

In conclusion, the genome of *P. multocida* strain P6 exhibits a dynamic and mosaic architecture shaped by horizontal gene transfer and diverse mobile genetic elements. The strain harbors a broad repertoire of metabolic features, including carbohydrate-active enzyme systems and secondary metabolite biosynthesis pathways, which may enhance its adaptability across different environmental niches. The coexistence of CRISPR–Cas systems with genomic islands and prophages reflects ongoing interactions between genome defense and horizontal gene acquisition. The identified antimicrobial resistance gene repertoire and conserved virulence-associated gene content indicate that strain P6 represents an environmentally adapted lineage with genetic features potentially relevant to infection and persistence. Together, these findings expand current knowledge of *P. multocida* genomic diversity and evolution and provide a descriptive genomic framework for future studies with expanded sampling aimed at elucidating host adaptation and disease-associated traits.

The statistically significant enrichment in mobilome-associated genes (COG X) distinguishes P6 from reference strains and may underlie its enhanced capacity for horizontal gene acquisition and genomic adaptation. The comprehensive glycosyltransferase repertoire, particularly the sialylation-related enzymes operating alongside iron acquisition systems and unique TonB-dependent receptors, indicates sophisticated mechanisms for immune evasion and metabolic adaptation to diverse host environments. Future research should focus on experimental validation of selected predicted virulence-associated functions (e.g., capsule/LOS-associated loci and iron acquisition systems), investigation of regulatory networks affecting surface glycan expression, and expanded regional surveillance of *P. multocida* in sheep to better understand the distribution of mobile genetic elements and antimicrobial resistance determinants.

## Data Availability

The original contributions presented in this study are included in the article/Supplementary Material. Further inquiries can be directed to the corresponding author.

## Supplementary Materials

The following supporting information can be downloaded at: https://www.mdpi.com/article/10.3390/microorganisms14010154/s1, Table S1: Source and origin of the *Pasteurella multocida* strains used in this study; Table S2: Characteristics of the CRISPR-Cas loci identified in strain P6; Table S3: Genetic composition of genomic island GI01 in *Pasteurella multocida* P6; Table S4: Genetic composition of prophage regions Ph01 and Ph02 in *Pasteurella multocida* P6; Table S5: Summary of transposon elements identified in *Pasteurella multocida* P6; Table S6: Summary of secondary metabolite biosynthetic gene clusters identified in strain P6; Table S7: Annotation of carbohydrate-active enzymes (CAZymes) in strain P6 genome; Table S8: Putative pathogenicity-related genes identified in strain P6 by BLASTP against the PHI-base database; Table S9: Phenotypic classification of virulence-associated genes in strain P6 based on PHI-base mutant phenotypes; Table S10: Functional categorization of pathogenicity-associated genes in strain P6; Table S11: Proteins with predicted N-terminal signal peptides in strain P6; Table S12: Core components of the Sec-SRP, Tat, and partial T6SS protein secretion systems identified in strain P6; Table S13: High-confidence core virulence determinants in strain P6; Table S14: Comparative analysis of virulence-associated genes shared between *Pasteurella multocida* P6 and reference strain PM70 (AE004439); Table S15: Comparative analysis of antibiotic resistance genes (ARGs) in *Pasteurella multocida* strains P6 and PM70 (AE004439); Table S16: Comparative statistics of COG functional categories between *Pasteurella multocida* P6 and PM70 (AE004439); Table S17: Significantly differentiated COG categories between Pasteurella multocida P6 and PM70 (AE004439); Table S18: Comparative KEGG functional profiling of Pasteurella multocida P6 and PM70 (AE004439) across hierarchical levels.
